# The German Translation of the Stress and Anxiety to Viral Epidemics-9 (SAVE-9) Scale: Results from Healthcare Workers during the Second Wave of COVID-19

**DOI:** 10.3390/ijerph18179377

**Published:** 2021-09-05

**Authors:** Julia König, Seockhoon Chung, Verena Ertl, Bettina K. Doering, Hannah Comtesse, Johanna Unterhitzenberger, Antonia Barke

**Affiliations:** 1 Department of Clinical and Biological Psychology, Catholic University of Eichstaett-Ingolstadt, Ostenstraße 25, 85072 Eichstaett, Germany; Verena.Ertl@ku.de (V.E.); Bettina.Doering@ku.de (B.K.D.); hannah.comtesse@ku.de (H.C.); Johanna.Unterhitzenberger@ku.de (J.U.); Antonia.Barke@ku.de (A.B.); 2 Department of Psychiatry, Asan Medical Center, University of Ulsan College of Medicine, 88 Olympic-ro 43-gil, Songpa-gu, Seoul 05505, Korea

**Keywords:** COVID-19, SAVE-9, healthcare workers, work stress, anxiety, Germany

## Abstract

Healthcare workers (HCW) are among those most directly affected by the COVID-19 pandemic. Most research with this group has used ad hoc measures, which limits comparability across samples. The Stress and Anxiety to Viral Epidemics-9 scale (SAVE-9) is a nine-item scale first developed in Korea, and has since been translated into several languages. We report on data collected from 484 German HCW between November 2020 and March 2021, during the “second wave” of coronavirus infections. We conducted item analysis, confirmatory factor analysis on the previously found factor solutions of the SAVE-9, examined correlations with established measures of depression, generalized anxiety, and insomnia, and compared scores between different groups of HCW. The psychometric properties of the German SAVE-9 were satisfactory and comparable to previous findings from Korea and Russia. Correlations with mental health measures were positive, as expected. We found some significant differences between groups of HCW on the SAVE-9 which were consistent with the literature but did not appear on the other mental health measures. This suggests that the SAVE-9 taps into specifically work-related stress, which may make it a helpful instrument in this research area.

## 1. Introduction

After more than 18 months, the COVID-19 pandemic is far from over. The burden caused by the disease itself and the measures taken to curb its spread are felt in all of society. Healthcare workers (HCW) are at the forefront of dealing with the pandemic; they share in the burdens borne by everyone (e.g., curfews, restrictions in activities), but in addition, face specific stressors due to their profession (e.g., increased risk of infection, increased workload). Even though some researchers did not find evidence that more exposure to COVID-19 patients conferred a higher risk of infection in this group [1,2,3,4,5], there is strong evidence for markedly increased rates of COVID-19 in HCW compared to the general public [6,7,8,9,10,11,12,13,14].

### 1.1. Mental Health of German HCW during the Pandemic

While there is conflicting evidence of the infection risk in HCW relative to the general population, a clearer picture emerges with respect to mental health. Reviews show significant mental health problems in HCW during pandemics in general [15,16] and COVID-19 in particular [17,18,19,20]. Evidence from a German study of HCW demonstrates that the majority of respondents reported feeling “moderately” (50.2%) or “strongly” (23.4%) threatened by the pandemic, and that about half of the respondents felt a “negative” (48.3%) or “very negative” (5.4%) influence of the pandemic on their mood [21]. Nurses reported feeling more threatened than doctors and females more threatened than males. Kramer et al. [22] corroborated these negative effects of the pandemic on German HCW: Participants rated their subjective mental stress as high, worried strongly about the well-being of their families, and experienced marked fear of catching the virus and, in turn, infecting others. They did not, however, report reduced sleep quality or reduced time in their personal lives. Nurses scored significantly higher on items on subjective burden and subjective mental stress than doctors and other HCW. Concerning the prevalence of mental health disorders, a cross-sectional study of HCW [23] reported that 19.0% had probable depression and likewise 19.0% had probable clinical generalized anxiety. While cross-sectional studies cannot establish a causal link between the pandemic and mental well-being, they suggest that COVID-19 could be a negative influence on mental health in HCW.

### 1.2. The Stress and Anxiety to Viral Epidemics—9 Questionnaire (SAVE-9)

Many of the above-mentioned studies have used ad hoc questionnaires developed for the respective study only. This limits comparability between different samples and impedes progress of research in this important area. There is an urgent need for psychometrically sound questionnaires with validated translations. To address this problem, the Stress and Anxiety to Viral Epidemics-9 scale (SAVE-9) was introduced by Chung et al. [24] in a Korean and English version. The SAVE-9 is a brief measure that contains 9 items assessing fear of COVID-19 and stress in the workplace resulting from the pandemic. It has been translated into Albanian, Arabic, Chinese, French, German, Hausa, Italian, Japanese, Persian, Portuguese, Russian, Spanish, Thai, Turkish, and Traditional Chinese [25]. In the present paper, we validate the German version and compare findings to the results reported in previous studies.

In the Korean validation sample of 1019 HCW [24], the SAVE-9 demonstrated a good internal consistency (Cronbach’s α = 0.80) and validity, as indicated by the expected positive correlations with measures of generalized anxiety (Generalized Anxiety Disorder Scale, GAD-7; [26]) and depression (depression subscale of the Brief Patient Health Questionnaire, PHQ-9, [27]). An exploratory factor analysis yielded two factors. The first, labelled ‘anxiety about the viral epidemic’ (items 1, 2, 3, 4, 5, 8) related to fears regarding COVID-19. The second, labelled ‘work-related stress’, concerns issues such as a more negative attitude towards one’s job and fears of being avoided by others (items 6, 7, and 9). A receiver operating characteristic (ROC) analysis showed that the SAVE-9 had a good ability to discriminate between respondents experiencing no and at least some generalized anxiety (area under curve, AUC = 0.748). Using a score of ≥5 GAD-7, which denotes at least mildly elevated anxiety, as the criterion, the optimal cut-off for the SAVE-9 was ≥22, resulting in a sensitivity of 0.72 and a specificity of 0.61.

Mosolova et al. [28] validated a Russian translation of the SAVE-9. They also report a satisfactory internal consistency (Cronbach’s α = 0.79), and a good discriminatory ability (AUC = 0.808, sensitivity = 0.68~0.73, specificity = 0.76~0.80) at a cut-off of ≥18. However, a principal component analysis resulted in somewhat different factors, with items 1 and 5 changing over to the other factor. This implies that in this sample, the SAVE-anxiety factor contained specific fears of infection for oneself (items 2–4), friends and family (item 8). The SAVE-work stress factor, in contrast, was somewhat less restricted to work-related issues. To our knowledge, the SAVE-9 scale has been used in three further publications [29,30,31], but these did not focus on the psychometrics of the scale.

The aim of our study is to analyse the psychometric characteristics of the German SAVE-9 scale, to confirm the original factor structure, and to determine a cut-off score with the help of the GAD-7 [32], as has been done in previous publications and compare our results to data reported from different countries. We also compare the scores obtained by different groups of HCW. 

## 2. Materials and Methods

### 2.1. Procedure

The study is a cross-sectional online survey aimed at healthcare workers. The questionnaire was hosted on the platform Qualtrics (Qualtrics, Provo, UT, United States) and was online from November 2020 to March 2021. We recruited via professional societies, word of mouth, posts in social media, and posters and flyers distributed in hospitals. The study was approved by the ethics committee of the Catholic University of Eichstätt-Ingolstadt (KU). The participants received full study information and provided informed consent before proceeding to the anonymous online questionnaire. As an incentive for participation, participants were able to choose one of three charitable organizations to whom EUR 5 was to be donated for each completed survey. The money for these incentives was granted by the proFOR+ fund of the KU. We aimed to recruit German-speaking HCW over the age of 18 (inclusion criteria). Exclusion criteria were failure to agree with study participation and data use for research and answering less than one third of survey questions. 

### 2.2. Measures

Participants provided demographic and job-related information and completed the SAVE-9, as well as widely used measures of anxiety, depression, and sleep problems. At the end of the survey, they could leave additional comments.

Demographic and job-related information. Participants provided information on age, gender, profession and current function within the healthcare system and the city where they worked.

Stress and Anxiety to viral epidemics. The Stress and Anxiety to Viral Epidemics—9 Items (SAVE-9) was developed in Korea by Chung et al. [24] to measure the reactions of healthcare workers to the COVID-19 pandemic. Items are scored on a Likert-type scale ranging from 0 (never) to 4 (always). We used the German version of the SAVE-9 after translation and reverse-translation process. The German version is available online free of charge [25]. 

Anxiety. The Generalized Anxiety Disorder Scale-7 (GAD-7, [26], German version [32]) is a well-validated screening instrument for general anxiety. Its seven items are scored on a Likert-type scale from 0 (not at all) to 3 (almost every day). It has good internal consistency, Cronbach’s α = 0.89 [32]. The intensity of anxiety symptoms can be classified as minimal (0–4), mild (5–9), moderate (10–14), or severe (15–21 points).

Depression. To screen for depression, we used the German translation [33] of the PHQ-9 [27,34]. Respondents indicate agreement with the nine items on a four-point Likert-type scale ranging from 0 (not at all) to 3 (almost every day). Sum scores can be classified into five groups, healthy (0–4), subthreshold or mild depression (5–9), moderate depression (10–14), pronounced depression (15–19), and severe depression (20–27). Gräfe et al. [35], among others, found good psychometric properties in a sample of patients from general practitioners and internal medicine outpatient clinics, for the cut-off of ≥10, sensitivity = 0.95, specificity = 0.83, Cronbach’s α = 0.88. 

Sleep problems. The Insomnia Severity Index (ISI; [36]) is a 7-item insomnia scale. Items are rated on a 5-point Likert-type scale ranging from 0, denoting no difficulties, to 4, denoting severe difficulties. The German version of the ISI [37,38] has a good internal consistency, 0.76 ≤ Cronbach’s α ≤ 0.86 in four samples. Dieck et al. [37] report an excellent ability of the measure to discriminate between good and poor sleepers using a cut-off of >10 (sensitivity = 91.4, specificity = 84.4). 

### 2.3. Statistical Analyses

Given the shortness of the instruments used, we only included participants in each analysis who had no missing values on the scales in question; therefore, sample sizes vary slightly. To analyse the psychometric properties of the German SAVE-9, we conducted a standard item analysis, including item difficulties and item-total correlations, determined the internal consistency (Cronbach’s α) and conducted confirmatory factor analyses (CFA) of the different two-factor solutions found in previous research. We computed the χ^2^/df ratio, root-mean-square error of approximation (RMSEA, <0.05 very good fit, 0.05–0.08 good fit, [39]), the comparative fit index CFI (>0.95 very good fit, [40]) and the standardized root-mean-square residual SRMR (≤0.08 acceptable fit, [40]). Following Chung et al. [24], we conducted an ROC analysis using the GAD-7 (cut-off ≥ 5) to examine the ability of the SAVE-9 to discriminate between individuals experiencing no vs. some anxiety-related distress, and to determine an optimum cut-off. We also analysed correlations of the sum scores between the different symptom measures. We compared correlation coefficients according to Eid et al., 2011 [41]. We also compared our results to those found in previous samples with the same measures. We further explored differences in SAVE-9 and the other symptom scales between different groups of participants (i.e., doctors, nurses, and other HCW, males and females) with *t*-test or analyses of variance (ANOVA) followed by post hoc tests as appropriate. We used IBM SPSS (IBM Corp., Armonk, New York, USA) Statistics for Windows, Version 26 and IBM SPSS Amos, Version 25 for analyses. 

## 3. Results

### 3.1. Participants

Of 538 responses where participants indicated agreement with participation and data use, we excluded 42 who had completed less than one third of the survey. As the SAVE-9 was developed for use in the healthcare field, we also deleted 12 responses indicating the person did not work in healthcare. Our final sample included 484 respondents. The vast majority were German (478 or 98.8%), but there were 3 Austrian and 3 Swiss participants whom we kept in the sample because German is the native language of the areas where they worked. The sample contained respondents from all 16 German federal states. Moreover, large cities, as well as smaller ones and rural regions, were represented. Demographic data and some further information are included in Table 1 and mental health questionnaire results in Table 2. 

### 3.2. SAVE-9

#### 3.2.1. Item Analysis and Internal Consistency

Corrected item-scale correlations ranged from r_itc_ = 0.229 to r_itc_ = 0.580 with a mean of r_itc_ = 0.428. The most difficult item (i.e., the least endorsed) was item 7, “After this experience, do you think you will avoid treating patients with viral illness?” (p_i_ = 0.166) and the least difficult (i.e., most endorsed) item 8, “Do you worry your family or friends may become infected because of you?” (p_i_ = 0.623); the mean item difficulty was p_i_ = 0.434. Further details are shown in Table 3. Internal consistency of the whole scale was corrected Cronbach’s α = 0.744; removing item 1 would have improved the internal consistency of the scale to α = 0.751 (Table 3).

#### 3.2.2. Factor Structure

The confirmatory factor analysis of the two factors found in the original publication yielded a very good fit, χ^2^/df ratio = 3.06, CFI = 0.933, RMSEA = 0.065 (90% CI 0.049–0.082), SRMR = 0.049. However, the solution found in the Russian sample obtained even better fit indices, χ^2^/df ratio = 1.92, CFI = 0.970, RMSEA = 0.044 (90% CI 0.025–0.062), SRMR = 0.035. Therefore, this is also the preferred solution for the German SAVE-9. 

#### 3.2.3. Cut-Off

As done by Chung et al. [24], we conducted a ROC analysis to determine the cut-off for the SAVE-9 using a GAD-7 score of 5 or more as the criterion. The area under curve (AUC) was 0.787, and we determined 14 as the optimal cut-off, yielding a specificity of 66.8% and a sensitivity of 75.3%. 

#### 3.2.4. Comparison to International Samples

The SAVE-9 showed positive correlations with the other three symptom measures in the moderate to high range. Spearman-Rho correlation coefficients are given in Table 4. 

For ease of comparison, we give the relevant scores of the different samples in which the SAVE-9 has been tested in Table 5. The SAVE-9 scores in our sample were markedly lower than those reported by Chung et al. [24], t(1469) = 14.7127 *p* < 0.001, and Lee et al. [29], t(888) = 11.8239, *p* < 0.001, but higher than those reported by Mosolova et al. [28], t(1567) = 3.5051, *p* < 0.001. 

#### 3.2.5. SAVE-9 Scores in Different Groups of HCW

To learn more about the SAVE-9 and its subscales, we compared scores between different groups (type of healthcare worker, gender, experience with COVID-19 patients, own COVID-19 infection) using independent sample *t*-tests or one-way ANOVAs as appropriate (Table 6). The SAVE-9 and SAVE-work stress scales differed between different types of HCW, F(2, 474) = 4.516, *p* = 0.011 and F(2, 474) = 7.523, *p* = 0.001, respectively. Post hoc tests revealed that nurses scored significantly higher than doctors, while other healthcare professionals did not differ from either group. A similar pattern emerged for direct experience with COVID-19: the SAVE-9 and both its subscales were significantly higher for HCW who had direct experience of working with COVID-19 patients and their relatives and those who had not, SAVE-9 T(464) = 3.293, *p* = 0.001, SAVE-anxiety T(464) = 2.185, *p* = 0.029, SAVE-work stress T(464) = 3.400, *p* = 0.001. In addition, the full scale and the work stress subscale (but not SAVE-9 anxiety) showed higher scores in those who had been infected and quarantined than those who had not, SAVE-9 T(51.003) = 2.386, *p* = 0.021, SAVE-work stress T(52.801) = 2.706, *p* = 0.009. 

## 4. Discussion

The German translation of the SAVE-9 showed satisfactory psychometric properties, which were comparable to the Russian and original Korean samples. The cut-off for at least somewhat elevated stress (>14) was lower than the cut-offs reported previously (18 and 22, respectively; [24,28]). Regarding the factor structure of the German SAVE-9, the structure proposed by Molosova et al. (2020) demonstrated a better fit to the data than the original structure proposed by Chung et al. (2020) and was therefore adopted. In comparison to the Korean factor structure, this makes the anxiety subscale more specific to COVID-19 fear, and the work stress subscale somewhat less specific to the workplace. In accordance with Mosolova et al. [28], we consider the original names of the subscales still suitable even with these two changes. 

In the item analyses, most items showed satisfactory characteristics with the slight exception of item 1 “Are you afraid the virus outbreak will continue indefinitely?”, which showed low item-whole correlations and detracted from the overall internal consistency of the scale. If this had been a new development, we would have considered excluding it from the scale. However, in order to preserve the comparability with other published studies, we chose to retain the item nevertheless. It is possible that this is due to issues with the translation, or rather, difficulties in correctly translating subtle emotional meanings. The English “to be afraid” can mean a slight concern (e.g., “I am afraid we are out of coffee, would you like something else?”) or a real fear (“I am afraid of heights.”) and the German translation is somewhat closer to the first meaning, possibly eliciting a prediction about the pandemic rather than an emotional response. It is possible that the Korean version was more frequently interpreted according to the second meaning, which also fits with the item belonging to the “anxiety” factor in the Korean sample.

As expected, SAVE-9 scores showed positive correlations in the moderate range with the other symptom scales, and correlations were comparable in magnitude with those found previously. While the psychometric data and intercorrelations of mental health questionnaires were comparable to previous samples, the level of the reported stress, as expressed in the mean scores, differed. The pattern observed in our sample seems more similar to the one reported by Mosolova et al. [28], with our sample exhibiting higher scores in both SAVE-9 and GAD-7. A possible reason for this difference may be the timing of the two studies. Mosolova et al. [28] collected their data in the spring of 2020, when the pandemic had been widely known and discussed for only a few weeks. We, on the other hand, collected our data between November 2020 and March 2021, after pandemic conditions had applied for several months [42]. It seems likely that HCW experienced demoralization and cumulative stress due to the long duration of the pandemic. Therefore, timing may explain why both SAVE-9 and GAD-7 scores were more than one point higher in our sample. 

In contrast, SAVE-9 scores in our sample were significantly lower than the Korean ones, while GAD-7 and PHQ-9 were markedly higher (in fact, the mean GAD-7 score was twice as high in the German sample) Timing of the studies alone cannot account for this, however, it might be an effect of the differences between the two countries’ public health policies. South Korea has been very successful in limiting the spread of COVID-19 with a “test, trace, isolate” strategy [43] and has imposed less strict social distancing measures than Germany. The populations of the two countries are roughly comparable, at 77 million in Korea and 83 million in Germany. During the data collection phases of both Korean studies [24,29], South Korea had infection rates below 400 new confirmed cases each week [44] and it is possible that the burden of the pandemic really became apparent mostly as increased work stress in HCW who had to adapt to the new hygiene protocols, conduct tests, and work with infected patients. Conversely, Germany went through its second wave of infections during our data collection phase, with 50,000 to over 173,000 new confirmed cases each week [45], and social distancing measures of varying intensity, including curfews, school closings, and others [42]. These non-work-related stressors, caused by both the countermeasures and the infection and death rates, may account for the high mental health burden in our respondents, possibly while attenuating their appraisal of the specifically work-related stressors. 

Even though GAD-7, PHQ-9, and ISI scores did not differ between different groups of HCW, we were able to detect some differences in the SAVE-9. HCW who had worked directly with COVID-19 patients and/or their relatives scored significantly higher on the total scale and both subscales than those who had not, indicating that this group experienced more COVID-19 related stress, but not more general mental health problems. Nurses scored higher than doctors on the total scale and work stress subscale. This is in accordance with previous research which also tended to show a higher burden in nurses (e.g., [15,17,22]) and indicates that the SAVE-9 possibly measures this aspect better than the other mental health scales. The same, though only for the total scale and work stress subscale, was true for HCW who had been infected and quarantined. These results strongly suggest that the SAVE-9 taps into the specific pandemic-related stress experienced by HCW rather than “only” their general mental health, thereby adding valuable information, with the work stress subscale possibly performing better in this respect than the anxiety subscale. Having a measure for specifically work-related stress among HCW may be helpful in examining factors or interventions contributing to or alleviating such stress without it being confounded with the general mental health burden connected with the overall pandemic situation. Given that SAVE-9 and GAD-7 therefore seem to measure different (though related) concepts, this implies that the cut-off, which was determined using a GAD-7 as a criterion, may need further investigation. We would encourage future researchers to revisit the question of a useful cut-off, and, in the meantime, report means and standard deviations as being possibly more helpful than percentages below and above cut-off.

The study has several limitations. The sample is a convenience sample of HCW who self-selected for participation in the study and is therefore not representative. The sample is also smaller than those used in previous studies. However, we were able to attain data from HCW from a variety of institutions and all areas of Germany, with many participants working directly with COVID-19 patients. Importantly, all data are cross-sectional and therefore our analyses allow for no causal interpretation. Our sample reported having been infected with COVID-19 at a rate of 9.1%. The estimated rate of infections nationwide at the time our survey ended was 3.1%, assuming 83,190,556 inhabitants [46] and 2,575,849 known infections [47]. This is about a threefold increased risk as compared to the general population. Given the conflicting evidence of infection rates in HCW compared to the general population, it is not clear whether persons with COVID-19 infections were over-represented in our sample. The decision of choosing a GAD-7 score of ≥5 as a criterion for a cut-off, which was made in order to replicate the original study, can be considered problematic as the GAD-7 is a measure of generalized anxiety and is shorter than the SAVE-9. In future, it would be preferable to compare the SAVE-9 with other measures of COVID-19-related stress. 

## 5. Conclusions

Even given the limitations, our results point toward the SAVE-9 being an economical, valid, and psychometrically sound instrument to measure pandemic-related stress in HCW. Having such an instrument is valuable for comparing national and international research, and validated questionnaires should replace the ad hoc instruments frequently used to date. 

## Figures and Tables

**Table 1:** Sample description.

Variable	Category	n	%
Gender	female	389	80.4
	male	92	19.0
Age group	<20	10	2.1
	20–29	119	24.6
	30–39	130	26.9
	40–49	92	19.0
	50–59	99	20.5
	60+	32	6.6
Marital status	married	217	44.8
	in relationship	152	31.4
	single	94	19.4
	other	19	3.9
Type of HCW ^1^	Nursing staff	241	49.8
	Doctor	97	20.0
	Other	144	29.8
Workplace	General hospital	240	49.6
	Specialized hospital	73	15.1
	University hospital	53	11.0
	Outpatient clinic/doctor’s office	84	17.4
	Other	26	5.4
Direct experience with COVID-19 patients	yes	365	75.4
	no	106	21.9
Has been infected with COVID-19	yes	44	9.1
	no	427	88.2
Lifetime depression, anxiety, or insomnia	yes	154	31.8
	no	317	65.5
Do you need help for your mental health?	yes	111	22.9
	no	350	72.3

^1^ HCW = healthcare worker.

**Table 2:** Mental health questionnaire data.

Scale	M (SD)	n (%)	Cronbach’s α
SAVE-9 (*n* = 479)	15.68 (5.60)		0.744
GAD-7 (*n* = 475)	7.50 (4.73)		0.863
	minimal anxiety (0–4)	139 (29.3)	
	mild anxiety (5–9)	199 (41.9)	
	moderate anxiety (0–14)	93 (19.6)	
	severe anxiety (>14)	44 (9.3)	
PHQ-9 (*n* = 468)	7.74 (5.20)		0.863
	no/subthreshold depression (0–9)	319 (68.2)	
	moderate depression (10–14)	94 (20.1)	
	marked depression (15–19)	39 (8.3)	
	severe depression (20–27)	16 (3.4)	
ISI (*n* = 467)	9.58 (6.14)		0.863
	no insomnia (0–10)	264 (56.5)	
	insomnia (>10)	203 (43.5)	

Note. SAVE-9 Stress and Anxiety to Viral Epidemics—9 items; GAD-7 Generalized Anxiety Disorder Scale—7 (range 0–21); PHQ-9 Patient Health Questionnaire depression subscale (range 0–27); ISI Insomnia Severity Index (range 0–28).

**Table 3:** Item analysis (n = 479).

Nr	Item	M (SD)	Item Difficulty	Corrected Item-Scale Correlation	Squared Multiple Correlation	Cronbach’s Alpha without Item	Factor [15]	Factor [19] and Current Sample
1	Are you afraid the virus outbreak will continue indefinitely?	2.36 (1.06)	0.589	0.229	0.066	0.751	anxiety	work stress
2	Are you afraid your health will worsen because of the virus?	1.77 (0.98)	0.440	0.536	0.392	0.704	anxiety	anxiety
3	Are you worried that you might get infected?	2.14 (0.97)	0.531	0.580	0.475	0.697	anxiety	anxiety
4	Are you more sensitive towards minor physical symptoms than usual?	1.89 (1.11)	0.472	0.349	0.167	0.733	anxiety	anxiety
5	Are you worried that others might avoid you even after the infection risk has been minimized?	1.49 (1.13)	0.370	0.398	0.193	0.725	anxiety	work stress
6	Do you feel sceptical about your job after going through this experience?	1.41 (1.23)	0.351	0.424	0.228	0.721	work stress	work stress
7	After this experience, do you think you will avoid treating patients with viral illness?	0.67 (0.92)	0.166	0.384	0.182	0.727	work stress	work stress
8	Do you worry your family or friends may become infected because of you?	2.51 (1.03)	0.623	0.566	0.388	0.697	anxiety	anxiety
9	Do you think that your colleagues would have more work to do due to your absence from a possible quarantine and might blame you?	1.45 (1.29)	0.360	0.385	0.167	0.729	work stress	work stress

**Table 4:** Intercorrelations (Spearman’s rho) between symptom measures.

Scale	SAVE-9 F1	SAVE-9 F2	GAD-7	PHQ-9	ISI
SAVE-9	0.833 **	0.862 **	0.566 **	0.524 **	0.461 **
SAVE-9 anxiety		0.459 **	0.419 **	0.372 **	0.357 **
SAVE-9 work stress			0.529 **	0.511 **	0.433 **
GAD-7				0.778 **	0.624 **
PHQ-9					0.747 **

Note. SAVE-9 Stress and Anxiety to Viral Epidemics—9 items; F1 = factor 1 anxiety; F2 = factor 2 work stress; GAD-7 Generalized Anxiety Disorder Scale—7; PHQ-9 Patient Health Questionnaire depression subscale; ISI Insomnia Severity Index. ** *p* < 0.001.

**Table 5:** Different samples where SAVE-9 was used.

Study	[15]	[20]	[22]	[19]	This Study
N	1019	406		1090	484
Country	Korea	Korea	Italy	Russia	Germany
	M (SD)	M (SD)	M ^a^	M (SD)	M (SD)
SAVE-9	20.3 (5.7)	20.1 (5.5)	14.1	14.47 (6.58)	15.68 (5.60)
GAD-7	3.7 (4.0)			6.34 (5.75)	7.50 (4.73)
PHQ-9	5.0 (4.6)	4.9 (4.3)			7.74 (5.20)

Note. SAVE-9 Stress and Anxiety to Viral Epidemics—9 items GAD-7 Generalized Anxiety Disorder Scale—7 PHQ-9 Patient Health Questionnaire depression subscale ^a^. The authors do not give means and standard deviations in their paper, so we calculated the overall mean from the data given. This was not possible for the standard deviation.

**Table 6:** Comparisons of mental health indicators between different groups.

Group	SAVE-9	SAVE-Anxiety	SAVE-Work Stress	GAD-7	PHQ-9	ISI
	M (SD)	M (SD)	M (SD)	M (SD)	M (SD)	M (SD)
Type of healthcare worker						
Doctors	14.19 (5.52) ^a^	7.88 (2.81)	6.30 (3.50) ^b^	7.54 (4.74)	7.13 (5.08)	9.05 (5.78)
Nurses	16.19 (5.39) ^a^	8.31 (3.16)	7.88 (3.18) ^b^	7.61 (4.67)	7.90 (4.95)	9.81 (6.14)
Other	15.82 (5.84) ^a^	8.58 (3.08)	7.24 (3.68) ^b^	7.31 (4.87)	7.91 (5.71)	9.58 (6.40)
Gender						
Female	15.76 (5.56)	8.37 (3.07)	7.39 (3.43)	7.54 (4.78)	7.80 (5.18)	9.68 (6.12)
Male	15.22 (5.81)	7.96 (3.11)	7.26 (3.51)	7.28 (4.57)	7.45 (5.36)	9.08 (6.28)
Experience with Covid-19 patients or their relatives						
Yes	16.15 (5.63)	8.48 (3.18)	7.67 (3.39)	7.65 (4.70)	8.01 (5.29)	9.82 (6.20)
No	14.12 (5.29)	7.73 (2.70)	6.39 (3.46)	7.18 (5.29)	6.99 (4.93)	9.03 (5.98)
Covid-19 infection and quarantine						
Yes	17.64 (5.88)	8.93 (3.14)	8.70 (3.42)	6.93 (4.08)	8.07 (5.21)	10.07 (6.39)
No	15.43 (5.46)	8.19 (3.00)	7.34 (3.41)	7.50 (4.71)	7.64 (5.12)	9.48 (6.01)

(^a,b^) Doctors showed lower scores than nurses and other HCW, who did not differ from each other. Values in italics are significantly different from the values in the other group. Note. SAVE-9 Stress and Anxiety to Viral Epidemics—9 items; GAD-7 Generalized Anxiety Disorder Scale—7; PHQ-9 Patient Health Questionnaire depression subscale; ISI Insomnia Severity Index.

## Data Availability

The dataset is not publicly available because participants did not provide consent to this. It is, however, available from the first author upon reasonable request.
